# Supplementary material for: A unified model framework for the multi-attribute consistent periodic vehicle routing problem
Source: PLoS One. 2020 Aug 3;15(8):e0237014. doi: 10.1371/journal.pone.0237014 (PMC7398502; doi:10.1371/journal.pone.0237014)
Supplement: S1 Appendix — It was applied to evaluate the effect of the inclusion of each of the additional constraints (28), (30), and (31) on the gap. (PDF) [file pone.0237014.s001.pdf]

Paired t-test

Same F1 + (28) or +(30)

30	f1	f1 + 31
Mean	100	100
Variance	0	0
Observations	6	6
Pearson correlation coeff	#¡DIV/0!	
Hypothetic mean differen	0	
degrees of freedom	5	
t statistic	#¡DIV/0!	
P(T<=t) one tail	#¡DIV/0!	
Critical t (one tail)	#¡DIV/0!	
P(T<=t) two tails	#¡DIV/0!	
Critical t (two tails)	#¡DIV/0!	

Paired t-test

30	f2	f2 + 31
Mean	71.05	69.9583
Variance	67.911	19.2463
Observati	6	6
Pearson c	0.669995	
Hypotheti	0	
degrees o	5	
t statistic	0.429773	
P(T<=t) or	0.342626	
Critical t (	2.015048	
P(T<=t) tw	0.685252	
Critical t (	2.570582	

Paired t-test

30	f3	f3 + 31
Mean	20.2617	19.05
Variance	251.909	277.832
Observati	6	6
Pearson c	0.97614	
Hypotheti	0	
degrees o	5	
t statistic	0.815	
P(T<=t) or	0.22606	
Critical t (	2.01505	
P(T<=t) tw	0.45213	
Critical t (	2.57058	

Paired t-test

30	f2	f2 + 28
Mean	71.05	69.935
Variance	67.911	106.283
Observati	6	6
Pearson c	0.4211	
Hypotheti	0	
degrees o	5	
t statistic	0.26958	
P(T<=t) or	0.39913	
Critical t (	2.01505	
P(T<=t) tw	0.79826	
Critical t (	2.57058	

Paired t-test

30	f2	f2 + 30
Mean	71.05	69.185
Variance	67.911	37.8003
Observati	6	6
Pearson c	0.85916	
Hypotheti	0	
degrees o	5	
t statistic	1.05781	
P(T<=t) or	0.16927	
Critical t (	2.01505	
P(T<=t) tw	0.33855	
Critical t (	2.57058	

Paired t-test

20	f1	f1 + 31
Mean	89.83333	100
Variance	620.1667	0
Observations	6	6
Pearson correlation coeff	#¡DIV/0!	
Hypothetic mean differen	0	
degrees of freedom	5	
t statistic	-1	
P(T<=t) one tail	0.181609	
Critical t (one tail)	2.015048	
P(T<=t) two tails	0.363217	
Critical t (two tails)	2.570582	

Paired t-test

20	f2	f2 + 31
Mean	31.83167	32.3333
Variance	36.15002	41.8667
Observati	6	6
Pearson c	0.993724	
Hypotheti	0	
degrees o	5	
t statistic	-1.470815	
P(T<=t) or	0.100652	
Critical t (	2.015048	
P(T<=t) tw	0.201305	
Critical t (	2.570582	

Paired t-test

	f3	f3 + 31
Mean	10.47	3.88833
Variance	150.989	90.7148
Observati	6	6
Pearson c	0.47843	
Hypotheti	0	
degrees of freedom		
t statistic	1.4155	
P(T<=t) or	0.10804	
Critical t (	2.01505	
P(T<=t) tw	0.21608	
Critical t (	2.57058	

Paired t-test

20	f2	f2 + 28	
Mean	20.35	14.5	
Variance	496.375	504.7	
Observati	6	6	5
Pearson c	0.9082		
Hypotheti	0		
degrees o	5		
t statistic	1.49448		
P(T<=t) or	0.09764		
Critical t (	2.01505		
P(T<=t) tw	0.19528		
Critical t (	2.57058		

Paired t-test

20	f2	f2 + 30
Mean	20.35	38
Variance	496.375	1548.4
Observati	6	6
Pearson c	0.28603	
Hypotheti	0	
degrees o	5	
t statistic	-1.10053	
P(T<=t) or	0.16062	
Critical t (	2.01505	
P(T<=t) tw	0.32124	
Critical t (	2.57058	

Paired t-test

60	f1	f1 + 30
Mean	100	97.18333
Variance	0	47.60167
Observations	6	6
Pearson correlation coeff	#¡DIV/0!	
Hypothetic mean differen	0	
degrees of freedom	5	
t statistic	1	
P(T<=t) one tail	0.181609	
Critical t (one tail)	2.015048	
P(T<=t) two tails	0.363217	
Critical t (two tails)	2.570582	

Paired t-test

60	f2	f2 + 31
Mean	91.54432	89.1732
Variance	16.85085	103.615
Observati	6	6
Pearson c	0.97766	
Hypotheti	0	
degrees o	5	
t statistic	0.932886	
P(T<=t) or	0.19685	
Critical t (	2.015048	
P(T<=t) tw	0.393701	
Critical t (	2.570582	

Paired t-test

60	f3	f3 + 31
Mean	31.105	30.29
Variance	172.403	265.828
Observati	6	6
Pearson c	0.96644	
Hypotheti	0	
degrees o	5	
t statistic	0.4038	
P(T<=t) or	0.35152	
Critical t (	2.01505	
P(T<=t) tw	0.70305	
Critical t (	2.57058	

Paired t-test

60	f2	f2 + 28
Mean	91.5443	90.2677
Variance	16.8508	23.7979
Observati	6	6
Pearson c	0.92425	
Hypotheti	0	
degrees o	5	
t statistic	1.64088	
P(T<=t) or	0.08087	
Critical t (	2.01505	
P(T<=t) tw	0.16175	
Critical t (	2.57058	

Paired t-test

60	f2	f2 + 30
Mean	91.5443	87.5791
Variance	16.8508	24.0995
Observati	6	6
Pearson c	0.91036	
Hypotheti	0	
degrees o	5	
t statistic	4.70612	
P(T<=t) or	0.00265	
Critical t (	2.01505	
P(T<=t) tw	0.00531	
Critical t (	2.57058	

f1 All = 100

Paired t-test

40	f2	f2 + 31
Mean	78.4583	74.652
Variance	18.3474	20.5678
Observati	6	6
Pearson c	0.877377	
Hypotheti	0	
degrees o	5	
t statistic	4.24341	
P(T<=t) or	0.004071	
Critical t (	2.015048	
P(T<=t) tw	0.008143	
Critical t (	2.570582	

Paired t-test

40	f3	f3 + 31
Mean	23.8833	22.5033
Variance	105.538	80.671
Observati	6	6
Pearson c	0.8869	
Hypotheti	0	
degrees o	5	
t statistic	0.71201	
P(T<=t) or	0.25415	
Critical t (	2.01505	
P(T<=t) tw	0.50829	
Critical t (	2.57058	

Paired t-test

40	f2	f2 + 28
Mean	78.4583	78.3004
Variance	18.3474	25.9622
Observati	6	6
Pearson c	0.77481	
Hypotheti	0	
degrees o	5	
t statistic	0.11945	
P(T<=t) or	0.45479	
Critical t (	2.01505	
P(T<=t) tw	0.90957	
Critical t (	2.57058	

Paired t-test

40	f2	f2 + 30
Mean	78.4583	80.0547
Variance	18.3474	29.1727
Observati	6	6
Pearson c	0.64113	
Hypotheti	0	
degrees o	5	
t statistic	-0.92539	
P(T<=t) or	0.19861	
Critical t (	2.01505	
P(T<=t) tw	0.39723	
Critical t (	2.57058	

f1 All = 100

Paired t-test

20B	f2	f2 + 31
Mean	12.81637	10.4516
Variance	673.7147	535.201
Observati	6	6
Pearson c	0.998507	
Hypotheti	0	
degrees o	5	
t statistic	1.854675	
P(T<=t) or	0.061404	
Critical t (	2.015048	
P(T<=t) tw	0.122808	
Critical t (	2.570582	

Paired t-test

20B	f3	f3 + 31
Mean	5.875	6.30333
Variance	85.6571	99.7658
Observati	6	6
Pearson c	0.99957	
Hypotheti	0	
degrees o	5	
t statistic	-1.33548	
P(T<=t) or	0.11964	
Critical t (	2.01505	
P(T<=t) tw	0.23928	
Critical t (	2.57058	

Paired t-test

20B	f2	f2 + 28
Mean	12.8164	7.61097
Variance	673.715	347.561
Observati	6	6
Pearson c	0.99414	
Hypotheti	0	
degrees o	5	
t statistic	1.65791	
P(T<=t) or	0.07912	
Critical t (	2.01505	
P(T<=t) tw	0.15823	
Critical t (	2.57058	

Paired t-test

20B	f2	f2 + 30
Mean	12.8164	10.7782
Variance	673.715	529.66
Observati	6	6
Pearson c	0.9988	
Hypotheti	0	
degrees o	5	
t statistic	1.57232	
P(T<=t) or	0.08834	
Critical t (	2.01505	
P(T<=t) tw	0.17668	
Critical t (	2.57058	

F1 All values equal

12	f1	f1 + 31
Mean	33.33333	33.33333
Variance	2666.667	2666.667
Observations	6	6
Pearson correlation coeff	1	
Hypothetic mean differen	0	
degrees of freedom	5	
t statistic	#¡DIV/0!	
P(T<=t) one tail	#¡DIV/0!	
Critical t (one tail)	#¡DIV/0!	
P(T<=t) two tails	#¡DIV/0!	

Paired t-test

12	f2	f2 + 31
Mean	9.166667	0
Variance	504.1667	0
Observati	6	6
Pearson c	#¡DIV/0!	
Hypotheti	0	
degrees o	5	
t statistic	1	
P(T<=t) or	0.181609	
Critical t (	2.015048	
P(T<=t) tw	0.363217	

F3 All = 0

Paired t-test

12	f2	f2 + 28
Mean	9.16667	0
Variance	504.167	0
Observati	6	6
Pearson c	#¡DIV/0!	
Hypotheti	0	
degrees o	5	
t statistic	1	
P(T<=t) or	0.18161	
Critical t (	2.01505	
P(T<=t) tw	0.36322	

Paired t-test

12	f2	f2 + 30
Mean	9.16667	0
Variance	504.167	0
Observati	6	6
Pearson c	#¡DIV/0!	
Hypotheti	0	
degrees o	5	
t statistic	1	
P(T<=t) or	0.18161	
Critical t (	2.01505	
P(T<=t) tw	0.36322	

Critical t (two tails)	#¡DIV/0!

Critical t (t	2.570582

Critical t (t	2.57058

Critical t (t	2.57058

FI All = 100

Paired t-test

16	f2	f2 + 31
Mean	45.07167	40.4311
Variance	1222.993	1077.09
Observati	6	6
Pearson c	0.989717	
Hypotheti	0	
degrees o	5	
t statistic	2.139124	
P(T<=t) or	0.042706	
Critical t (	2.015048	
P(T<=t) tw	0.085412	
Critical t (	2.570582	

Paired t-test

16	f3	f3 + 31
Mean	8.47	5.60167
Variance	89.8385	78.5237
Observati	6	6
Pearson c	0.87888	
Hypotheti	0	
degrees o	5	
t statistic	1.54326	
P(T<=t) or	0.09171	
Critical t (	2.01505	
P(T<=t) tw	0.18342	
Critical t (	2.57058	

Paired t-test

16	f2	f2 + 28
Mean	45.0717	39.4867
Variance	1222.99	1186.09
Observati	6	6
Pearson c	0.90529	
Hypotheti	0	
degrees o	5	
t statistic	0.90518	
P(T<=t) or	0.20343	
Critical t (	2.01505	
P(T<=t) tw	0.40687	
Critical t (	2.57058	

Paired t-test

16	f2	f2 + 30
Mean	45.0717	37.0882
Variance	1222.99	1141.99
Observati	6	6
Pearson c	0.97576	
Hypotheti	0	
degrees o	5	
t statistic	2.55264	
P(T<=t) or	0.02555	
Critical t (	2.01505	
P(T<=t) tw	0.0511	
Critical t (	2.57058	

FI : All = 100

Paired t-test

20	f2	f2 + 31
Mean	56.075	60.0147
Variance	1065.801	177.065
Observati	6	6
Pearson c	0.933154	
Hypotheti	0	
degrees o	5	
t statistic	-0.464233	
P(T<=t) or	0.330996	
Critical t (	2.015048	
P(T<=t) tw	0.661992	
Critical t (	2.570582	

Paired t-test

20	f3	f3 + 31
Mean	14.965	14.89
Variance	157.743	152.568
Observati	6	6
Pearson c	0.9984	
Hypotheti	0	
degrees o	5	
t statistic	0.25039	
P(T<=t) or	0.40612	
Critical t (	2.01505	
P(T<=t) tw	0.81225	
Critical t (	2.57058	

Paired t-test

20	f2	f2 + 28
Mean	56.075	43.095
Variance	1065.8	1294.98
Observati	6	6
Pearson c	0.91537	
Hypotheti	0	
degrees o	5	
t statistic	2.19397	
P(T<=t) or	0.03985	
Critical t (	2.01505	
P(T<=t) tw	0.0797	
Critical t (	2.57058	

Paired t-test

20	f2	f2 + 30
Mean	56.075	49.9819
Variance	1065.8	1017.38
Observati	6	6
Pearson c	0.97921	
Hypotheti	0	
degrees o	5	
t statistic	2.25354	
P(T<=t) or	0.03697	
Critical t (	2.01505	
P(T<=t) tw	0.07395	
Critical t (	2.57058	
