# Supplementary material for: A unified model framework for the multi-attribute consistent periodic vehicle routing problem
Source: PLoS One. 2020 Aug 3;15(8):e0237014. doi: 10.1371/journal.pone.0237014 (PMC7398502; doi:10.1371/journal.pone.0237014)
Supplement: S2 Appendix — It was applied to evaluate the significance of the four factors considered in the experimental design: objective function, centrality, frequency, and number of visits. (PDF) [file pone.0237014.s002.pdf]

General Factorial Regression: GapA versus Blocks, VisitsA, FreqA, CentA, OFA

Factor Information

Factor	Levels	Values
VisitsA	2	20, 30
FreqA	3	1.00, 1.33, 2.00
CentA	2	0, 1
OFA	3	1, 2, 3

Analysis of Variance

Source	DF	Seq SS	Contribution	Adj SS	Adj MS	F-Value	P-Value
Model	13	106139	92.26%	106139	8164.6	53.18	0.000
Blocks	1	30	0.03%	30	30.1	0.20	0.659
Linear	6	97241	84.53%	97241	16206.9	105.57	0.000
VisitsA	1	8313	7.23%	8313	8313.4	54.15	0.000
FreqA	2	1792	1.56%	1792	895.9	5.84	0.005
CentA	1	1255	1.09%	1255	1255.3	8.18	0.006
OFA	2	85881	74.65%	85881	42940.3	279.72	0.000
2-Way Interactions	6	8868	7.71%	8868	1478.0	9.63	0.000
VisitsA*FreqA	2	1762	1.53%	1762	881.0	5.74	0.005
VisitsA*OFA	2	5980	5.20%	5980	2990.2	19.48	0.000
CentA*OFA	2	1126	0.98%	1126	562.8	3.67	0.032
Error	58	8904	7.74%	8904	153.5		
Total	71	115043	100.00%				

Model Summary

S	R-sq	R-sq(adj)	PRESS	R-sq(pred)
12.3901	92.26%	90.53%	13721.0	88.07%

Regression Equation

```
GapA = 52.64 - 10.75 VisitsA_20 + 10.75 VisitsA_30 + 2.79 FreqA_1.00 + 4.22 FreqA_1.33
       - 7.01 FreqA_2.00 + 4.18 CentA_0 - 4.18 CentA_1 + 44.82 OFA_1 - 5.59 OFA_2
       - 39.22 OFA_3 - 1.70 VisitsA*FreqA_20 1.00 + 6.73 VisitsA*FreqA_20 1.33
       - 5.03 VisitsA*FreqA_20 2.00 + 1.70 VisitsA*FreqA_30 1.00 - 6.73 VisitsA*FreqA_30
1.33
       + 5.03 VisitsA*FreqA_30 2.00 + 8.20 VisitsA*OFA_20 1 - 12.71 VisitsA*OFA_20 2
       + 4.51 VisitsA*OFA_20 3 - 8.20 VisitsA*OFA_30 1 + 12.71 VisitsA*OFA_30 2
       - 4.51 VisitsA*OFA_30 3 - 1.63 CentA*OFA_0 1 - 3.81 CentA*OFA_0 2
+ 5.45 CentA*OFA_0 3
       + 1.63 CentA*OFA_1 1 + 3.81 CentA*OFA_1 2 - 5.45 CentA*OFA_1 3
```

General Factorial Regression: GapB versus Blocks, VisitsB, FreqB, Cent, OF

Factor Information

Factor	Levels	Values
VisitsB	4	12, 20, 40, 60
FreqB	3	1.00000, 1.33333, 2.00000
Cent	2	0, 1
OF	3	1, 2, 3

Analysis of Variance

Source	DF	Seq SS	Contribution	Adj SS	Adj MS	F-Value	P-Value
Model	25	234664	87.55%	234664	9386.6	33.18	0.000
Blocks	1	327	0.12%	327	326.6	1.15	0.285
Linear	8	197524	73.69%	197524	24690.5	87.27	0.000
VisitsB	3	91111	33.99%	91111	30370.4	107.35	0.000
FreqB	2	12940	4.83%	12940	6469.8	22.87	0.000
Cent	1	952	0.36%	952	952.2	3.37	0.069
OF	2	92521	34.52%	92521	46260.6	163.52	0.000
2-Way Interactions	16	36813	13.73%	36813	2300.8	8.13	0.000
VisitsB*FreqB	6	9639	3.60%	9639	1606.4	5.68	0.000
VisitsB*OF	6	21499	8.02%	21499	3583.2	12.67	0.000
FreqB*OF	4	5676	2.12%	5676	1418.9	5.02	0.001
Error	118	33383	12.45%	33383	282.9		
Total	143	268047	100.00%				

Model Summary

S	R-sq	R-sq(adj)	PRESS	R-sq(pred)
16.8198	87.55%	84.91%	49714.9	81.45%

Regression Equation

```
GapB = 45.95 - 33.31 VisitsB_12 - 15.05 VisitsB_20 + 20.63 VisitsB_40 + 27.73 VisitsB_60
       + 11.81 FreqB_1.00000 - 0.41 FreqB_1.33333 - 11.40 FreqB_2.00000 + 2.57 Cent_0
       - 2.57 Cent_1 + 31.13 OF_1 - 0.17 OF_2 - 30.96 OF_3 + 13.47 VisitsB*FreqB_12
1.00000
       - 12.23 VisitsB*FreqB_12 1.33333 - 1.24 VisitsB*FreqB_12 2.00000
       + 5.07 VisitsB*FreqB_20 1.00000 + 6.10 VisitsB*FreqB_20 1.33333
       - 11.18 VisitsB*FreqB_20 2.00000 - 10.14 VisitsB*FreqB_40 1.00000
       + 1.69 VisitsB*FreqB_40 1.33333 + 8.45 VisitsB*FreqB_40 2.00000
       - 8.40 VisitsB*FreqB_60 1.00000 + 4.43 VisitsB*FreqB_60 1.33333
       + 3.97 VisitsB*FreqB_60 2.00000 - 10.44 VisitsB*OF_12 1 - 7.88 VisitsB*OF_12 2
       + 18.32 VisitsB*OF_12 3 + 12.96 VisitsB*OF_20 1 - 19.10 VisitsB*OF_20 2
       + 6.14 VisitsB*OF_20 3 + 2.29 VisitsB*OF_40 1 + 10.14 VisitsB*OF_40 2
       - 12.43 VisitsB*OF_40 3 - 4.82 VisitsB*OF_60 1 + 16.84 VisitsB*OF_60 2
       - 12.03 VisitsB*OF_60 3 + 11.11 FreqB*OF_1.00000 1 - 2.47 FreqB*OF_1.00000 2
       - 8.63 FreqB*OF_1.00000 3 - 1.67 FreqB*OF_1.33333 1 - 1.65 FreqB*OF_1.33333 2
       + 3.32 FreqB*OF_1.33333 3 - 9.44 FreqB*OF_2.00000 1 + 4.12 FreqB*OF_2.00000 2
       + 5.32 FreqB*OF_2.00000 3
```

Equation averaged over blocks.
